# Cerebrospinal Fluid IL-12p40, CXCL13 and IL-8 as a Combinatorial Biomarker of Active Intrathecal Inflammation

**DOI:** 10.1371/journal.pone.0048370

**Published:** 2012-11-30

**Authors:** Bibiana Bielekova, Mika Komori, Quangang Xu, Daniel S. Reich, Tianxia Wu

**Affiliations:** 1 Neuroimmunology Branch, National Institute of Neurological Disorders and Stroke, National Institutes of Health, Bethesda, Maryland, United States of America; 2 Clinical Neurosciences Program, National Institute of Neurological Disorders and Stroke, National Institutes of Health, Bethesda, Maryland, United States of America; University of Texas at San Antonio, United States of America

## Abstract

Diagnosis and management of the neuroinflammatory diseases of the central nervous system (CNS) are hindered by the lack of reliable biomarkers of active intrathecal inflammation. We hypothesized that measuring several putative inflammatory biomarkers simultaneously will augment specificity and sensitivity of the biomarker to the clinically useful range. Based on our pilot experiment in which we measured 18 inflammatory biomarkers in 10-fold concentrated cerebrospinal fluid (CSF) derived from 16 untreated patients with highly active multiple sclerosis (MS) we selected a combination of three CSF biomarkers, IL-12p40, CXCL13 and IL-8, for further validation.

Concentrations of IL-12p40, CXCL13 and IL-8 were determined in a blinded fashion in CSF samples from an initial cohort (n = 72) and a confirmatory cohort (n = 167) of prospectively collected, untreated subjects presenting for a diagnostic work-up of possible neuroimmunological disorder. Diagnostic conclusion was based on a thorough clinical workup, which included laboratory assessment of the blood and CSF, neuroimaging and longitudinal follow-up. Receiver operating characteristic (ROC) curve analysis in conjunction with principal component analysis (PCA), which was used to combine information from all three biomarkers, assessed the diagnostic value of measured biomarkers.

Each of the three biomarkers was significantly increased in MS and other inflammatory neurological disease (OIND) in comparison to non-inflammatory neurological disorder patients (NIND) at least in one cohort. However, considering all three biomarkers together improved accuracy of predicting the presence of intrathecal inflammation to the consistently good to excellent range (area under the ROC curve = 0.868–0.924).

Future clinical studies will determine if a combinatorial biomarker consisting of CSF IL-12p40, CXCL13 and IL-8 provides utility in determining the presence of active intrathecal inflammation in diagnostically uncertain cases and in therapeutic development and management.

## Introduction

Neuroimmunological diseases represent a broad spectrum of diverse diagnoses, most of which, with the exception of MS, are considered rare disorders. While the presence or absence of contrast-enhancing lesions (CEL) on the MRI imaging of the CNS have been successfully utilized as a biomarker of focal inflammatory activity, not all types of CNS inflammation are associated with opening of the blood brain barrier (BBB). Specifically, pathology studies demonstrated that inflammation, both focal [1] and diffuse (e.g., in the meninges) [2], [3], often remains highly active in some patients with progressive MS, even though CEL become increasingly uncommon. Similarly, when patients with an established diagnosis of neuroinflammatory conditions, such as CNS lupus, present with new but nonfocal neurological complaints, it is often very difficult to gauge the degree to which these symptoms are driven by an active inflammatory process. Consequently, clinicians often face a diagnostic and therapeutic dilemma in relationship to neuroinflammatory conditions.

Although noninvasive biomarkers are undoubtedly preferred, CSF has been traditionally collected during the diagnostic workup of neuroinflammatory diseases for the quantification of intrathecal immunoglobulin synthesis, measured as CSF IgG index and oligoclonal bands (OCB), and for assessment of CSF pleiocytosis. While both of these measurements are diagnostically useful, only CSF WBC count can respond rapidly to a change in the inflammatory process. Unfortunately, this response is often unpredictable and insensitive, probably because it represents a combination of the input (migration of inflammatory cells into CNS tissue) and output (retention vs. egress of inflammatory cells from CNS tissue), which may fluctuate greatly based on the evolution or phenotype of the immune response. On the other hand, CSF IgG index and OCB are established indicators of intrathecal humoral immunity that respond only slowly, if at all, to acute exacerbations of the intrathecal inflammatory process, as evidenced by their constancy during exacerbations and therapeutically-induced remissions of MS disease process [4], [5].

Therefore, we searched for complementary CSF biomarkers, focusing on soluble factors secreted by activated immune cells or CNS glia in response to inflammatory stimuli. In a pilot experiment, we measured 18 soluble factors (i.e. IL-6, IL-7, IL-8, IL-10, IL-12p40, IL-12p70, IL-17, IL-21, IL-23, granzyme B, IFN-γ, vascular endothelial growth factor (VEGF), oncostatin M, lymphotoxin-α (LT- α), tumor necrosis factor-α (TNF-α), CX3CL1, CCL19 and CXCL13) in 10-fold concentrated CSF collected from 16 untreated patients with highly active inflammatory MS [6]. Only IL-8, IL-12p40 and CXCL13 were detectable in the vast majority of patients. Therefore, we selected these three soluble factors for comprehensive evaluation and validation studies.

IL-12p40 is one of the subunits shared by two related cytokines, IL-12 and IL-23. IL-12p40 is produced almost exclusively by cells of the myeloid lineage, such as monocytes, macrophages, microglia and myeloid dendritic cells in response to varied inflammatory stimuli [7], [8]. However, IL-12p40 is produced in 5–100-fold excess in comparison to IL-12 or IL-23 [9], [10]. While IL-12p40 was originally considered either a biologically inactive molecule [11] or an antagonist of IL-12 and IL-23 signaling [12]–[15], emerging studies suggest that IL-12p40 may also have unique biological activity [16], [17]. Specifically, IL-12p40 acts as a chemoattractant for cells of myeloid lineage [18], [19], and IL-12p40 homodimer (i.e. IL-12p80) induces nitric oxide synthase and LT-α in microglial cells [20], [21] and activates CD8 T cells [22]. Finally, IL-12p40 mRNA has been found in autopsied MS lesions [23].

CXCL13, a B cell chemoattractant, has been consistently detected in the CSF of active MS patients [24]–[27] and can help predict whether patients with clinically isolated syndrome (CIS) will develop definite MS [28]. However, elevated CSF levels of CXCL13 have been also observed in many other CNS inflammatory conditions [29]–[32]. Like IL-12p40, CXCL13 is also produced predominantly by cells of myeloid lineage such as follicular dendritic cells, monocytes and macrophages [33], [34], but also by (malignant) B and T cells [35]–[38]. CXCL13 is widely expressed in the ectopic lymphoid follicles associated with many chronic inflammatory conditions [38]–[44], including MS [2], [45]. In fact, it is believed that CXCL13 plays an important role in the initiation and maintenance of the ectopic lymphoid tissue [46], [47].

IL-8 is an inflammatory chemokine, the main function of which is to attract and activate neutrophils, basophils and a subpopulation of lymphocytes, but it also has a strong angiogenic effect. It is produced by many different cell types, including monocytes, lymphocytes, granulocytes, fibroblasts, endothelial cells, astrocytes and different epithelial cells [48]. Not surprisingly, therefore, elevated CSF IL-8 levels have been observed in varied neuroinflammatory conditions [49]–[56].

To develop and validate combinatorial biomarker of intrathecal inflammation, we measured, in a blinded fashion, IL-12p40, CXCL13 and IL-8 in two independent, prospectively acquired cohorts of untreated patients with neuroinflammatory disorders and embedded non-inflammatory controls. In the larger, confirmatory cohort, we also assessed the relationship of these new CSF biomarkers to clinically accepted measures of CNS inflammation, specifically IgG index, OCB, CSF WBC count and MRI CEL.

## Methods

### Patients

The study was approved by the institutional review boards of the University of Cincinnati (for WMS cohort) and NINDS (for NIB cohort), and all participants provided written informed consent. Both cohorts were prospectively acquired under natural history protocols headed by the same principal investigator (PI; B.B.). The first cohort, WMS, was acquired at the Waddell Center for Multiple Sclerosis, University of Cincinnati, OH, between 2/2006 and 12/2007. The second cohort provided samples as part of a natural history protocol at the Neuroimmunology Branch (NIB), National Institute of Neurological Disorders and Stroke (NINDS), between 7/2008 and 4/2011. Because both protocols studied subjects who presented for diagnostic workup of a possible CNS neuroimmunological disorder CSF was collected when subjects were not receiving disease-modifying therapy (DMT). Although vast majority of enrolled subjects have not received any prior immunomodulatory therapies, some patients with progressive MS have been exposed to prior immunomodulatory therapies, such as interferon beta preparations, glatiramer acetate and monthly pulses of intravenously-administered solumedrol; however, they were off these therapies for a minimum of 3 months before the CSF collection. Diagnosis of MS was made based on the McDonald criteria [57]. Patients presenting with clinically isolated syndrome (CIS) were reclassified as having definite MS if they developed a second clinical symptom or fulfilled criteria of MRI dissemination in time. As such, patients who retained the CIS diagnosis did not progress to MS within a 1-year follow-up period. Alternative diagnoses were made based on clinical diagnostic tests and prospective follow-up. Because of low number of patients with secondary progressive MS (SP-MS) in both cohorts (N = 1 in WMS cohort and N = 8 in NIB cohort), we grouped together subjects with primary progressive MS (PP-MS) and SP-MS as “progressive MS” cohort. The demographic data and diagnostic categories for both cohorts are depicted in Table 1.

**Table 1:** Diagnostic and demographic data.

	F-test	RRMS	Progressive MS	CIS	NIND	OIND	Total
Pilot (WMS)*	df = 3						
N (female/male)		28 (25/3)	3 (0/3)	7 (6/1)	26 (20/6)	8 (7/1)	72 (58/14)
Average Age							
(SD)		38.0 (8.1)	54.0 (4.6)	43.2 (13.1)	43.9 (11.2)	46.6 (11.4)	42.3 (10.9)
Average EDSS (SD)	0.0004	1.8 (1.1)	3.7 (2.5)	0.8 (0.8)	0.8 (1.2)^a^	1.8 (0.6)	1.5 (1.3)
Average S-NRS (SD)	0.0006	91.4 (7.0)	81.7 (12.1)	97.6 (3.1)	97.1 (5.3)^a^ ^c^	88.7 (7.5)	93.0 (7.5)
Average IgG Index (SD)	<0.0001	1.4 (1.2)	1.3 (0.3)	0.5 (0.02)^a^	0.5 (0.1)^a^	0.5 (0.1)^a^	0.9 (0.9)
Confirmatory (NIB) df = 4							
N (female/male)		66 (39/27)	41 (20/31)	9 (5/4)	33 (27/6)	18 (6/12)	167 (97/70)
Average Age (SD)		39.5 (10.8)	52.6 (8.2)	41.3 (13.4)	48.1 (9.7)	41.5 (13.5)	45.1 (11.6)
Average EDSS (SD)	<0.0001	1.7 (1.4)^b^	5.2 (1.8)	1.0 (1.1)^b^	2.6 (2.2)^b^	2.4 (2.0)	2.8 (2.2)
Average S-NRS (SD)	<0.0001	92.0 (9.4)^b^	68.3 (15.4)	96.0 (6.9)^b^	90.5 (12.3)^b^	80.0 (14.4)	84.1 (16.1)
Average IgG Index (SD)	<0.0001	1.1 (0.9)	0.9 (0.5)	0.7 (0.3)^a^	0.5 (0.1)^a^ ^b^	0.8 (0.6)	0.9 (0.7)

Age and sex were considered as covariate (if p<0.1). Three subjects with progressive MS in WMS cohort were excluded from ANOVA or ANCOVA.

a p<0.05 vs. RR-MS;

b p<0.05 vs. Progressive MS;

c p<0.05 vs. OIND.

CSF from both cohorts was processed using identical procedures: CSF samples were placed on ice immediately after collection and centrifuged (300 g×10 min) within 15 min. Cell-free supernatant was prospectively coded, aliquotted and cryopreserved at −80°C (without the addition of protease inhibitors) until analysis. All analyses were performed blindly, and the diagnostic code was broken by the PI after the collection of all data was completed.

### Biomarker measurement

The details of the methodology, including assay, manufacturer, detection limits and intra-assay coefficients of variance are depicted in Table 2. When using concentrated CSF, samples were volume concentrated using 3 kDa Amicon Ultra-0.5 ml centrifugation filters (Millipore) to achieve 10× (500 µl to 50 µl) or analogously 4–5× concentrations. Although this concentration procedure yielded expected loss of small molecular weight (MW) proteins, because all selected analytes have MW higher than 10 kDa (i.e. CXCL13 MW = 10.1 kDa, IL-8 MW = 11.1 and IL-12p40 MW = 40 kDa), only minor, predictable loss of measured analytes occurred after selected volume concentration (Table S1). For the smallest of the measured analytes, CXCL13, 4× concentration enhanced detection limit on average 3.74 times and 10× concentration enhanced detection limit on average 8.41 times.

**Table 2:** Methodological details of biomarker measurements.

Protein	Assay type, manufacturer and catalogue number	CSF concentration^a^	Detection limit^b^	Coefficient of variance
Pilot (WMS) cohort				
IL-12p40	Cytometric bead assay (BD; Cat # 560154)	10×	2.1 pg/ml	0–27.9%
CXCL13	ELISA (R&D; Cat # DY801)	5×	25.0 pg/ml	0–7.5%
IL-8	Cytometric bead assay (BD, Cat # 558277)	10×	2.0 pg/ml	0–21.9%
Confirmatory (NIB) cohort				
IL-12p40	ELISA (Invitrogen; Cat # KHC0121	4×	2.3 pg/ml	0–27.9%
CXCL13	ELISA (R&D Systems; Cat # DY801)	1×	62.5 pg/ml	0–7.5%
IL-8	Cytometric bead assay (BD, Cat # 558277)	1×	19.5 pg/ml	0–21.9%
IL-12p70	Cytometric bead assay (BD, Cat # 558283)	1×	4.9 pg/ml	n/a^c^
IL-23	ELISA (Bender MedSystems; Cat # BMD 2023/3)	1×	31.3 pg/ml	n/a^c^

a If indicated, CSF was concentrated using Millipore Amicon Ultra 3 kDa filters.

b Only linear part of standard curve was used for quantification of protein; when concentrated CSF was used, detection limit is recalculated to reflect utilized concentration factor.

c Intra-assay coefficient of variance could not be calculated because all data were below the detection limit of the assay.

### MRI

Spin-echo and gradient-echo T1-weighted images were collected following intravenous administration of 0.1 mmol/kg gadopentetate dimeglumine (Magnevist; Berlex) on both 1.5T and 3T scanners (GE Medical Systems) using 8-channel phased-array head coils (Invivo). Three scans were performed over a 2-month period. An experienced neuroradiologist (D.S.R.) counted the number of CEL with reference to pre-contrast T1-weighted as well as T2-weighted (both fast-spin-echo and fluid-attenuated inversion recovery) and proton-density-weighted images. The number of CEL in each scan was recorded.

### Statistical analyses

Statistical analyses were performed using SAS version 9.2. Where concentrations of proteins were below the lower detection limit, the lower detection limit of the assay was used for calculating statistics. Inverse transformation (Lamda = −1 based on Box-Cox transformation) was applied to the three biomarkers CSF values to reduce non-normality. To assess differences in the CSF biomarkers between different diagnostic categories, analysis of covariance (ANCOVA) or analysis of variance (ANOVA) was utilized for 4 categories in the WMS cohort (NIND, OIND, CIS and RR-MS and 5 categories in the larger NIB cohort (NIND, OIND, CIS, RR-MS and progressive MS), with gender and age (if significant at p<0.1) as covariates. Scheffé's post-hoc test was used to determine inter-category differences.

The above analysis was also performed to examine differences in Expanded Disability Status Scale (EDSS), Scripps Neurological Rating Scale (S-NRS) and IgG-index between different diagnostic categories. These four measures were transformed by Box-Cox technique.

For analysis of clinical utility of inflammatory biomarkers, ANCOVA and ANOVA were used to examine association between biomarkers and broad diagnostic categories of diseases with (OIND and RR-MS) or without (NIND) intrathecal inflammation. Receiver operating characteristic (ROC) [58], [59] curve analysis was used to assess the accuracy of CSF biomarkers to identify patients with intrathecal inflammation. Since the three biomarkers were found to be correlated, principal component analysis (PCA) was performed to derive the first component (PCA1) as the combined test. For each biomarker separately, and for PCA1, the area under the curve (AUC) and the optimal cutoff with maximum sum of sensitivity and specificity were calculated. If age was found to be significantly correlated to biomarker values in logistic regression model, the AUC was adjusted for age.

## Results

**Figure 1:**
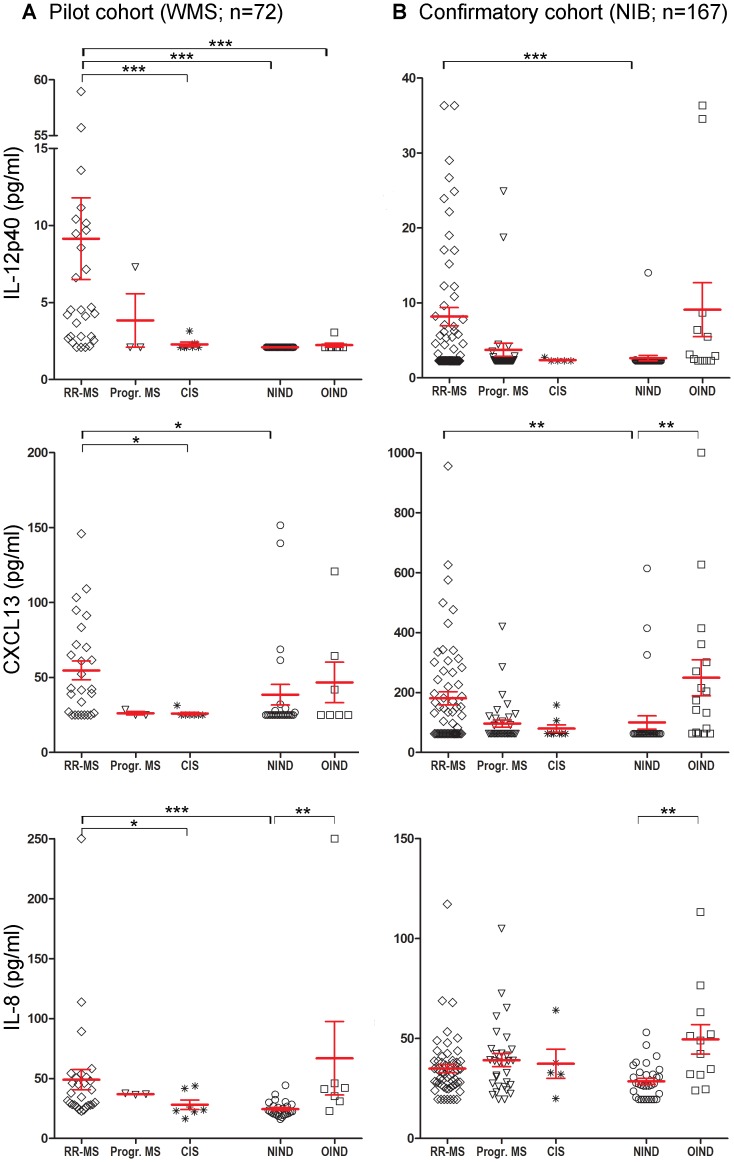
IL-12p40, CXCL13 and IL-8 CSF levels in patients with relapsing-remitting multiple sclerosis (RRMS), progressive multiple sclerosis (Prog-MS), clinically isolated syndrome (CIS), other inflammatory neurological diseases (OIND), and non-inflammatory neurological diseases (NIND) in the pilot (A) and confirmatory (B) cohorts. The average and standard deviations (SD) are included in scatter plots and the lower detection limit is indicated by the gray horizontal line in each plot. *P<0.05, **P<0.005 and ***P<0.0001.

Raw data from both cohorts, as well as means and standard deviations are depicted in Figure 1.

### Initial (WMS) cohort (Figure 1A)

In the smaller, WMS cohort (N = 72), we utilized identical methodology (including optimally concentrated CSF (Table 2)) as in our published pilot cohort of MS patients selected for their unusually high degree of CNS inflammatory activity [6]. In doing so, we wanted to assess the prevalence of selected biomarkers in the more representative, prospectively acquired cohort of subject who presented for the diagnostic workup of a possible neuroinflammatory CNS disorder.

After unblinding, RRMS patients were found to have a significantly higher concentration of CSF IL-12p40 (Mean 9.14 pg/ml; p<0.0001 compared to NIND, p = 0.004 compared to CIS), CXCL13 (Mean 54.72 pg/ml; p = 0.0215 compared to NIND, p = 0.0357 compared to CIS) and IL-8 (Mean 49.05 pg/ml; p<0.0001 compared to NIND, p = 0.0241 compared to CIS) than patients in the CIS (Means for IL-12p40: 2.29 pg/ml, CXCL13: 25.92 pg/ml and IL-8: 28.17 pg/ml) or NIND group (Means for IL-12p40: 2.10 pg/ml, CXCL13: 38.51 pg/ml and IL-8: 24.52 pg/ml). For CSF IL-12p40, MS patients also had significantly higher concentrations in comparison to OIND subjects (Mean 2.24; p = 0.005). Finally, OIND patients had significantly higher CSF IL-8 levels (Mean 66.86; p = 0.0035) in comparison to NIND subjects.

### Confirmatory (NIB) Cohort (Figure 1B)

A test that requires 10× concentration of the CSF (and thus 2 ml of CSF/test) is unlikely to gain broad clinical utility. Based on the results from the WMS cohort, we estimated that use of undiluted CSF for CXCL13 and IL-8 measurement would not significantly alter the results of utilized assays. Unfortunately, this was not true for IL-12p40. Therefore, in the larger NIB cohort (N = 167), we switched to a more sensitive ELISA IL-12p40 assay but still needed to implement a 4-fold concentration of the CSF in order to reach detection limit comparable to the one utilized in the WMS cohort (Table 2).

Using these updated assays, we observed that RRMS patients had higher levels of IL-12p40 (Mean 8.17 pg/ml; p = 0.0006) and CXCL13 (Mean 180.58 pg/ml; p = 0.0017), but not IL-8 (Mean 34.96 pg/ml), in comparison to NIND controls (Means for IL-12p40: 2.64 pg/ml, CXCL13: 100.13 pg/ml and IL-8: 28.61 pg/ml). No significant difference in any of the CSF biomarkers was observed between RRMS and OIND patients. For IL-8, the only statistically significant difference resided in higher CSF values in OIND controls (Mean 49.58; p = 0.002) in comparison to NIND subjects.

### Cytokine origin of CSF IL-12p40

Because IL-12p40 can be secreted as a monomer, a homodimer (IL-12p80) or a heterodimer with IL-12 (IL-12p70) or IL-23, we also measured IL-12p70 and IL-23 in patients who had detectable CSF levels of IL-12p40. As in the original pilot cohort [6] we could not detect IL-12p70 or IL-23 in any of the tested CSF samples. Thus, we conclude most of the measured CSF IL-12p40 represents either monomer or homodimer IL-12p80.

### Relationship between new and traditional CSF inflammatory biomarkers: IgG index, OCB and CSF WBC count

**Figure 2:**
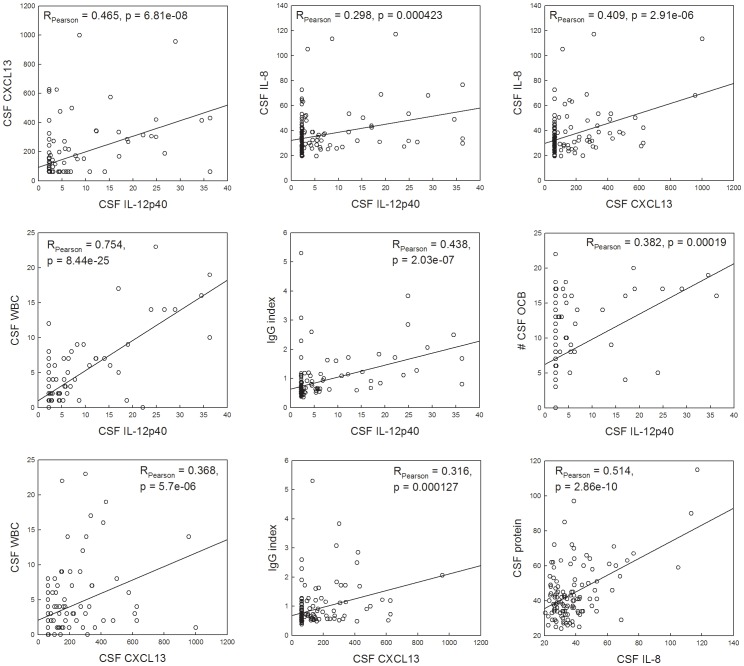
Correlations between CSF IL-12p40, CXCL13 and IL-8 (all measured in pg/ml) and traditional clinical measures of intrathecal inflammation: CSF biomarkers measured by NIH clinical laboratory: WBC count (# of cells per mm^3^ measured in unspun CSF), IgG index (normal range 0.26–0.62), OCB and total protein (g/dl) and CEL measured as described in detail in method section. Correlation coefficients and p values are detailed in each panel. The data originate from the confirmatory (NIB) cohort only.

All three selected biomarkers were significantly correlated with each other (Figure 2, upper raw panels). Additionally, IL-12p40 correlated strongly with CSF WBC count (Pearson r = 0.754, p<0.0001), moderately with IgG index (Pearson r = 0.438, p<0.0001) and mildly also with the number of OCB (Pearson r = 0.382, p = 0.00019; Figure 2, middle raw panels). Correlations of CXCL13 with traditional clinical biomarkers of intrathecal inflammation were overall less robust (with CSF WBC, Pearson r = 0.368, p<0.0001; with IgG index, Pearson r = 0.316, p = 0.000127 and with number of OCB, Pearson r = 0.254, p = 0.00961). Finally, IL-8 did not correlate with CSF WBC, IgG index or OCB after Bonferroni correction for multiple comparisons, but had instead moderate correlation to total CSF protein (Pearson r = 0.514, p<0.0001; Figure 2, lower raw panels).

### Relationship between CSF biomarkers and MRI CEL

We obtained three different measures of CEL. The first was the average number of gadolinium CEL on three consecutive monthly MRIs, which gave us a measure of overall disease activity around the time of LP. The second was the number of CEL in the MRI closest to the LP (5.3±5.7 days away for all patients), and the third was the number of CEL in the MRI that was performed closest to, but before, the LP (12.4±9.0 days before the LP for all patients). The third measure (i.e. CEL on MRI performed prior to the LP) was most strongly correlated to the CSF biomarkers in the entire NIB cohort (Spearman r = 0.518, p<0.0001 with IL-12p40, Spearman r = 0.374, p = 0.002 with CXCL13, and no significant correlations with IL-8 after Bonferroni adjustment for multiple comparisons). Considering only MS patients, we found significant positive associations between CSF levels of IL-12p40 (Spearman r = 0.559, p = 0.0002) and CXCL13 (Spearman r = 0.378, p = 0.0149), but not IL-8, with the third measure of CEL on brain MRI.

### Clinical utility of CSF biomarkers in identifying patients with intrathecal inflammation

In order to assess the clinical utility of the three biomarkers, we pooled together patients with RR-MS and OIND, two diagnostic categories with known intrathecal inflammation, and asked if selected biomarkers, individually or in combination, can differentiate them from NIND controls. We omitted in this analysis subjects with progressive MS because of uncertainty about which of them may have active intrathecal inflammatory process.

**Figure 3:**
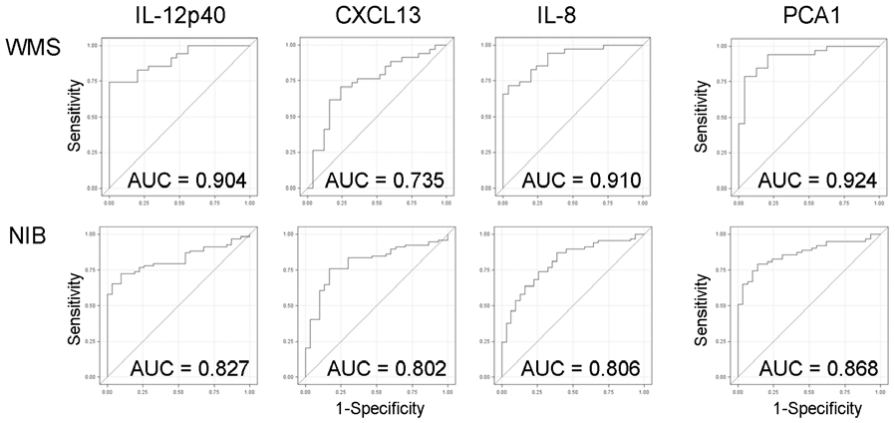
ROC curves for all three CSF biomarkers and their PCA1 for both WMS and NIB cohorts.

Because all three biomarkers were significantly correlated (Figure 2, upper panels), we also used linear combination of three biomarkers in PCA analysis. We observed that the first principal component (PCA1) explained over 60% of the variance in both cohorts. We evaluated diagnostic value (i.e. the ability to differentiate RR-MS or OIND from NIND subjects) of each biomarker separately and their PCA1 combination in gold standard ROC analysis, which ranks diagnostic value of the tests based on C-statistics (i.e. AUC values; Figure 3) and allows selection of an ideal operating point that provides an optimum trade-off between false-positive and false-negative results [60]. Thus, we calculated AUC values, 95% confidence intervals (CI), sensitivity, specificity and optimal cut-off values for all three biomarkers and for the PCA1 variable separately for both cohorts (Table 3). We observed that combining all three biomarkers into PCA1 consistently improved predictive accuracy of the test in comparison to each individual biomarker (Figure 3).

**Table 3:** Clinical utility of CSF biomarkers.

Protein	AUC*	95% CI	Optimal cut-off	Sensitivity	Specificity	False positive rate	False negative rate
Pilot (WMS) cohort							
IL-12p40	0.904	0.832–0.976	2.1 pg/ml	0.743	1.000	0	0.257
CXCL13	0.735	0.602–0.868	32.68 pg/ml	0.588	0.840	0.167	0.400
IL-8	0.910	0.840–0.980	27.42 pg/ml	0.829	0.760	0.240	0.171
PCA1 (63.4%)	0.924	0.874–0.997	n/a	0.879	0.833	0.121	0.167
Confirmatory (NIB) cohort							
IL-12p40	0.827	0.748–0.906	2.47 pg/ml	0.580	1.000	0	0.483
CXCL13	0.802	0.713–0.890	62.5 pg/ml	0.595	0.900	0.006	0.542
IL-8	0.806	0.717–0.895	27.98 pg/ml	0.710	0.613	0.197	0.513
PCA1 (64.3%)	0.868	0.796–0.940	n/a	0.762	0.862	0.077	0.375

* AUC is the percentage of randomly drawn pairs for which the test is correct (i.e. it correctly classifies the two patients in the pair).

## Discussion

While there are many studies that have reported elevated levels of different cytokines in the CSF of patients with MS and controls, few are consistently reproduced. In our pilot study utilizing 10 fold-concentrated CSF derived from 16 patients with highly active inflammatory MS, we found levels of IL-7, IL-12p70, IL-17, IL-21, IL-23, granzyme B, VEGF, oncostatin M, LT-α and TNF-α below detection limit of highly sensitive cytometric bead assay in 100% of the samples [6]. IL-6, IL-10, IFN-γ, CXCL13 and CCL19 were detectable only in a proportion of studied samples. IL-12p40, CXCL13 and IL-8 were most consistently expressed in untreated MS patients with active intrathecal inflammation and therefore were selected for further validation.

For the CXCL13, the CSF biomarker most extensively studied in MS field, our data fully reproduce published studies [24]–[28]. On the other hand, we believe that technical differences can explain the apparent discrepancy between our study and those that reported a more abundant cytokine profile in the CSF of MS patients. Delay in the CSF processing, leading to *in vitro* activation of the immune cells in the CSF sample, or release of the cellular content during cryopreservation of unspun CSF would all be expected to increase detection of soluble inflammatory biomarkers. In contrast, we focused on the detection of those factors that have been released into the CSF strictly *in vivo*, by placing CSF on ice immediately after collection and spinning the sample within 15 minutes of collection to remove all cells and debris. Second, we used only the linear part of the standard curve to derive results, which ensures that proteins are detected well above the noise of each assay. Because we were aware that many studies reported data close to, or below the published detection limits of utilized assays, we concentrated CSF to enhance the dynamic range of our assay. Lastly, all samples were processed using identical standardized procedures and biomarkers were measured in a blinded fashion, eliminating nonbiological differences that may occur due to different methods of CSF collection, processing and storage.

The strength of our study resides in the analysis of two independent cohorts of untreated patients with CNS inflammatory and non-inflammatory conditions, prospectively acquired by the same investigators. Relative under-representation of SP-MS and PP-MS subjects in these diagnostic cohorts precludes definite conclusions regarding the utility of measured biomarkers for progressive MS subtypes. While the samples were acquired during diagnostic work-up, patients were followed longitudinally for at least 1 year, which led to diagnostic conclusion in majority of studied subjects. We also view utilization of different commercially available assays and different CSF concentrations (Table 2) as a strength of our study, because it indicates that our results are robust and not dependent on specific methodology.

We found that newly added cytokine IL-12p40 had excellent specificity for intrathecal inflammation in both cohorts in comparison to much more extensively studied CXCL13 and IL-8. After unblinding, only one NIND patient in both cohorts (NIB 135; Figure 1B) was found to have high CSF IL-12p40 (14.04 pg/ml). Review of the data from the NIH clinical laboratory revealed that NIB 135, who carried a diagnosis of SLE, had CSF pleocytosis (7 white blood cells per microliter) and CSF specific OCB, both of which reflect intrathecal inflammation. This retrospective review indicated that NIB 135 was initially misclassified in our database and was included in OIND cohort for subsequent analyses. On the other hand, IL-8 was consistently more sensitive and less specific in comparison to both IL-12p40 and CXCL13.

While CXCL13 was found to have slightly lower sensitivity and specificity than IL-12p40 in both cohorts, mostly overlapping cells of origin and very similar correlation profile with traditional biomarkers of intrathecal inflammation indicates that IL-12p40 and CXCL13 measure analogous inflammatory pathways. A plausible explanation for why the association between CEL and CSF IL-12p40 or CXCL13 was strongest for the MRI that preceded the LP is that the opening of the blood-brain barrier (BBB) recruited a large number of blood-derived monocytes that became activated and released IL-12p40 and CXCL13 after myelin phagocytosis. Resident microglia may have contributed to IL-12p40 and CXCL13 secretion as well. This would suggest that IL-12p40 and CXCL13 do not participate in the events leading to BBB opening, which is supported by published observations that systemic administration of IL-12p40-targeting therapies does not abrogate development of CEL [61].

On the other hand, IL-8 seems to reflect different inflammatory pathway than IL-12p40 and CXCL13, as evidenced by lacking correlations with CSF WBC count, IgG index, OCB or CEL, but instead having moderate correlation with total CSF protein. Nevertheless, CSF IL-8 concentrations were consistently elevated in OIND patients and generally also elevated in RR-MS subjects, although reaching statistical significance only in WMS cohort. Furthermore, CSF IL-8 levels also correlated with IL-12p40 and CXCL13. As mentioned in the introduction, IL-8 is produced intrathecal by wide array of cell types, including abundant astrocytes. This may explain the high sensitivity but relatively low specificity of IL-8 for acute inflammatory process, because astroglial activation may be present also in noninflammatory (e.g. neurodegenerative) conditions. The failure of IL-8 to correlate with MRI measure of MS disease activity suggests that IL-8 is not pathophysiologically linked to the formation of focal MS lesions. This conclusion is supported by the data that daclizumab treatment, which results in profound inhibition of CEL, inhibits CSF levels of IL-12p40 [6], CXCL13 [62] but not of IL-8 [6] and by high CSF IL-8 levels in non-MS OIND subjects.

Our study fully supports the intuitive idea that combining several biomarkers that measure partially overlapping processes in the same pathogenic pathway can significantly enhance diagnostic accuracy of the test in comparison to the measurement of a single biomarker. While utilizing 4× concentrated CSF is still unpractical for broader clinical use, implementing novel methodologies, such as combination of multiplexing with improved signal detection through more sensitive antibody conjugates or detection systems should allow even today commercialization of this and analogous combinatorial biomarkers. Similarly, we expect that new, highly sensitive and quantitative methodologies, such as selected reaction monitoring mass spectrometry (SRM-MS) will make combinatorial biomarkers broadly applicable in future clinical practice.

In conclusion, our study indicates that measuring in parallel CSF IL-12p40, CXCL13 and IL-8 represents combinatorial biomarker of active intrathecal inflammation. We are currently prospectively testing (in the clinical trial of intrathecal rituximab in SP-MS; Clinicaltrials.gov identifier NCT01212094) whether this combinatorial biomarker has any predictive value in identify those patients with progressive MS that may have an active intrathecal inflammatory process amenable to immunomodulatory treatments. Similarly, it may find clinical utility in aiding and monitoring therapeutic decisions in patients with OIND.

## Supporting Information

Table S1 (DOC)
