# Supplementary material for: Cerebrospinal Fluid IL-12p40, CXCL13 and IL-8 as a Combinatorial Biomarker of Active Intrathecal Inflammation
Source: PLoS One. 2012 Nov 30;7(11):e48370. doi: 10.1371/journal.pone.0048370 (PMC3511462; doi:10.1371/journal.pone.0048370)
Supplement: Table S1 — (DOC) [file pone.0048370.s001.doc]

**Table S1**

|  | **Total protein** | | | | |  | **CXCL13** | | | | |
| --- | --- | --- | --- | --- | --- | --- | --- | --- | --- | --- | --- |
|  | **1x (pg/ml)** | **4x (pg/ml)** | **4x dilution factor^a^** | **10x (pg/ml)** | **10x dilution factor^a^** |  | **1x (pg/ml)** | **4x (pg/ml)** | **4x dilution factor^a^** | **10x (pg/ml)** | **10x dilution factor^a^** |
| **#1** | 758.0 | 1507.0 | 2.0 | 4582.0 | 6.0 |  | 20.0 | 41.0 | 2.1 | 182.0 | 9.1 |
| **#2** | 778.0 | 1272.0 | 1.6 | 4329.0 | 5.6 |  | 118.0 | 447.0 | 3.8 | 769.0 | 6.5 |
| **#3** | 825.0 | 2473.0 | 3.0 | 4308.0 | 5.2 |  | 19.0 | 83.0 | 4.4 | 166.0 | 8.7 |
| **#4** | 831.0 | 1634.0 | 2.0 | 4466.0 | 5.4 |  | 0.0 | 0.0 |  | 117.0 |  |
| **#5** | 662.0 | 1236.0 | 1.9 | 2507.0 | 3.8 |  | 0.0 | 0.0 |  | 48.0 |  |
| **#6** | 573.0 | 1414.0 | 2.5 | 2995.0 | 5.2 |  | 0.0 | 14.0 |  | 38.0 |  |
| **#7** | 659.0 | 1349.0 | 2.0 | 2685.0 | 4.1 |  | 0.0 | 0.0 |  | 20.0 |  |
| **#8** | 593.0 | 1380.0 | 2.3 | 2697.0 | 4.5 |  | 0.0 | 0.0 |  | 17.0 |  |
| **#9** | 757.0 | 1803.0 | 2.4 | 4594.0 | 6.1 |  | 0.0 | 20.0 |  | 74.0 |  |
| **#10** | 680.0 | 1961.0 | 2.9 | 3149.0 | 4.6 |  | 0.0 | 0.0 |  | 30.0 |  |
| **#11** | 717.0 | 1560.0 | 2.2 | 3762.0 | 5.2 |  | 43.0 | 205.0 | 4.8 | 400.0 | 9.3 |
| **Average** | **712.09** | **1659.67** | **2.25** | **3756.63** | **5.07** |  | **18.18** | **73.64** | **3.74** | **169.18** | **8.41** |
| **SD** | 86.76 | 378.01 | 0.42 | 867.58 | 0.74 |  | 35.91 | 138.33 | 1.20 | 228.41 | 1.29 |
|  |  |  |  |  |  |  |  |  |  |  |  |
| ^a^ 4x and 10x dilution factors were calculated based on comparison of measured concentration in 4x and 10x concentrated samples as compared to undiluted samples for analyte that were detectable in undiluted (i.e. 1x) CSF | | | | | | | | | | | |
